# Supplementary material for: Safe and effective thrombolysis in free flap salvage: Intra-arterial urokinase infusion
Source: PLoS One. 2023 Mar 13;18(3):e0282908. doi: 10.1371/journal.pone.0282908 (PMC10010546; doi:10.1371/journal.pone.0282908)
Supplement: Attachment — Submitted filename: Response to Reviewers.docx [file pone.0282908.s003.docx]

**We appreciate your comments and request to correct the manuscript. We believe that the valuable comments from the reviewers helped to improve our manuscript. We hope that the paper is now suitable for publication in *PLOS one.***

Reviewer #1: The study is complete and presented in an intelligible fashion. Data are consistent and results are correct. Statistical analysis has been provided in a correct way and also the surgical technique was accurate. We are perfectly agreeing to publication.

**ANSWER: We appreciate your kind comments.**

Reviewer #2: While your manuscript is interesting and presents potential, it presents some serious concerns which must be accounted for and addressed accordingly.

• None of the tables from 1 through 4 appear to be present in the current version of your submission. This is a big oversight: the data is crucial and needs to be assessed.
**ANSWER: We agree with you. Unfortunately, four tables were omitted while editing the manuscript to meet the format of PLOS one. We added them appropriately to the revised manuscript.**

• In Serletti’s study from the 1990s (ref. 14), they salvaged flaps with venous compromise by administering 250.000 IU of urokinase into the arterial recipient vessel proximally to the arterial anastomosis through the free flap, prior to 5000 IU of heparin administered intravenously, with no heparin post-operatively. They reported 8 free flaps with vascular compromise: 3 had evidence of arterial thrombosis, while 5 had venous compromise. They tested their protocol on the latter 5 cases. The aim of your study is to investigate “the effectiveness of intra-arterial urokinase infusion through free flap tissue to develop a protocol for re-exploration to prevent thrombotic failure.” However, the rationale for your study is not very clear: your study performed over two decades later presents a slightly higher patient population of free flap salvage cases (16 vs 5). Nevertheless, the differences which made your study design unique are not addressed sufficiently. Namely, the external venous drainage through the resected vein, the pressure infusion pump, the lower urokinase dosage. Was your aim the validation of a thrombolytic protocol for free flap salvage with strategies to reduce the risk of hemorrhagic events? Please clarify this in your abstract and at the end of your introduction.
**ANSWER: We appreciate your comment. As you know, if venous thrombosis occurs in the free flap, even if revision surgery is performed, the loss of flap, including partial necrosis, would be inevitable. Microsurgeons are all burdened with this catastrophic event, and the suggestion of a safe thrombolysis method would be welcome for all. It is helpful to use the urokinase like this study, but the expression of the systemic side effect using urokinase has been an obstacle to its use. This is because free flap surgery has many bleeding sites on the donor and recipient sites, so the use of verified heparin has always been a subject to pay attention. Therefore, as in this study, it is a great advantage that high-dose urokinase can be used in a short period of time without systemic circulation. You said that our urokinase is less used, but if we calculate our dose per hour, it is upto 600,000 IU/hr because infusion time is about 10 minutes. The use of more than 500,000 IU of urokinase per day may have a systemic side effect such as intracranial hemorrhage, hematochezia, and gross hematuria. Although Serletti did not mention side effects, the lack of a protocol for the urokinase since 1990s raises doubts about its use.**

**In other words, the advantage of this study is that high-dose urokinase can be used in a short period of time to perform thrombolysis in patients who have undergone free flap, without any systemic circulation. We emphasized this a little more with the red font in abstract and introduction.**

- **The present study investigates the effectiveness of high-dose intra-arterial urokinase infusion within a short period of time without systemic circulation through free flap tissue to develop a protocol for re-exploration to prevent thrombotic failure.**

• Your protocol was used just for the cases with venous compromise, which was 15 cases, regardless of whether arterial compromise was also present concomitantly or not (6 had arterial thrombosis). You state that 13 of 16 (81.3%) flaps survived completely, and just developed 2 partial necrosis? That’s 15. What happened to the 16th flap? The way you present your figures is very confusing. Just state how many out of the starting 581 flaps developed vascular compromise, how many of those were of venous origin and required salvage using your protocol.
**ANSWER: Thank you for the comments. Among 581 flaps, we have experienced much more thrombotic events than 16 cases included in this study. Meanwhile, we discovered the process of thrombolysis using urokinase, and we decided to publish the results because of the successful salvage of free flaps. Then, it could not be simply asserted that 16 cases out of 581.**

**One of case experienced arterial thrombosis with decreased venous drainage, but not venous thrombosis. So, we stated the targeted case as ‘venous thrombus or venous insufficiency’. In the revised version, we changed it to ‘**venous thrombus or venous insufficiency even if it was not thrombosis’ **with blue font in the Method and Discussion sections.**

**As for the number of flaps, there were 11 complete survival, 2 partial necrosis, and 3 total loss. Therefore, the expression was refined as follows to prevent the confusion in the Abstract and Results sections.**

**13 (81.3%) flaps survived completely, but 2 cases showed partial necrosis and secondary healing.**

- **13 (81.3%) flaps survived ~~completely~~, but 2 cases among them showed partial necrosis and secondary healing.**

• In your “Thrombolysis protocol” you mention that urokinase infusion was stopped “after the vein was sufficiently drained”. Could you define that with more precision? Was it enough to observe transparent fluid draining through the venous pedicle?
**ANSWER: As you supposed, it was judged that it was sufficient if transparent fluid drain at the same speed as venous blood drainage was observed. However, since it is not an expression limited to drain speed, it was written in front of it that the aspect of flap margin bleeding was also observed. Then, we changed the expression "sufficiently drained" to "****when venous drainage was observed at a sufficient rate." with brown font.**

• While Serletti did not administer any heparin post-operatively, you did so to prevent recurrent thrombus formation. Was the dosage adjusted according to patient weight? What was the ideal aPTT range you were seeking? How long is post-operative anticoagulation after free flap salvage maintained and is it correlated with what you routinely perform in your facility for systemic thromboembolic prophylaxis?

**ANSWER: We usually have administered heparin while keeping it in the aPTT range of 30 to 50 for 7 days. And it's a regimen that our hospital usually administers when a thrombotic tendency is suspected after a free flap. We added it under green shadow in Methods section.**

**For postoperative management, heparin was** **administered to prevent recurrent thrombus formation and adjusted while monitoring activated partial thromboplastin time (aPTT) 3 times a day.**

- **For postoperative management, heparin was administered to prevent recurrent thrombus formation for 7 days and adjusted while monitoring activated partial thromboplastin time (aPTT) range of 30 to 50 seconds 3 times a day.**

• You stated that your mean “time to re-exploration was 45.4 hours (range: 24–88 hours)”. As you know, Serletti et al. (ref. 14) conducted a study similar to yours in 1998. He used no flap monitoring strategy other than an external Doppler probe and clinical assessment. They reported a mean re-exploration time of 3.6 days. How would you explain the significant difference? Did you use any flap-monitoring technique?

**ANSWER: Thank you for the question. Of course, we have monitored free flap with hand-held Doppler and clinical signs. Additionally, we also use portable ultrasonography to determine the blood flow of pedicles. Since this checks the pedicle's blood flow, it may be a way to catch thrombolytic changes faster than monitoring with clinical sign and doppler signals alone. However, since this study is not about monitoring, it was not written in detail to write concisely.**

• Evidence has shown that the rate of flap salvage significantly decreased after 48 h. (PMID: 28648581) Were there any cases of late venous thrombosis (defined as beyond 72 hours)? There should be since the higher range of your mean time to re-exploration was 88 hours, but this could not be assessed due to the missing tables. If so, were they managed differently? Were the complications you reported correlated with time to re-exploration?

**ANSWER: We added 4 table in the revised manuscript. The mean time to re-exploration in this study was 45.4 ± 18.1 hours range of 24 to 88. Among them, one case was implemented within 24 hours, nine cases were implemented between 24 and 48 hours, five cases between 48 and 72 hours, and one case was more than 72 hours. Since revision surgery is performed under the judgment of surgeon that abnormalities are found during flap monitoring and surgery is necessary, it is difficult to determine the relationship between time to re-exploitation and complications.**

• In Serletti’s study, out of the 5 salvaged flaps, 2 developed marginal loss of tissue while one had some fat necrosis. That’s a 60% flap-related complication rate due to the prolonged ischemia. Why would you say that you only had 2 cases of partial flap necrosis while 13 flaps survived completely (i.e. 18.4% flap-related complications). How do you explain this difference?

**ANSWER: Thank you for the question. Serletti's study and this study differ in the number of flaps included and the protocol of thrombolysis, surgical method, and postoperative management are also different, so it is difficult to compare the complication rate. For two decades, it seems that there would have been accumulation of knowledge and advances in surgical methods and instruments.**

• In case of potential endothelial or mechanical damage from vessel handling, could you clarify which micro-anastomosis technique was used? Was it the posterior wall-first approach? Was heparinized solution used during the procedure? (PMID: 24706545)

**ANSWER: Thank you for the question. We emphasize the gentle manipulation of vessel during microsurgery is important.**

• The flaps used in reconstructive surgery are prone to ischemia and hypoxia, which imply a considerable risk of wound-healing complications. Hemodilution has been shown to improve tissue oxygenation in ischemic flaps. (PMID: 12170063) Was hemodilution taken into consideration when planning the flap? (PMID: 26313824; PMID: 26818324)

**ANSWER: Thank you for the question. However, we can’t agree with the necessity of hemodilution. Moreover, in clinical environment, hemodilution is difficult to apply in patients.**

**In our surgery, the ischemic time of free flap is usually less than 60 minutes. According to PMID: 9160132, ischemic time did not affect flap survival or partial loss within 3 hours.**

• Regarding your discussion, consider addressing more substantially the many differences with other studies such as Serletti et a. (ref. 14) and Nelson et al (ref. 16) compared to yours. Additionally, it should be stated how selective administration of thrombolytic agents to the free flap’s arterial circulation at lower dosages (100.000 IU) has already been shown in anecdotal evidence to prevent systemic complications, despite not having an external venous drainage. (PMID: 2810204) Additionally, your limitations section should address how future research should strive to obtain larger cohorts in the setting of a clinical trial to make this evidence more robust.

**ANSWER: Thank you for the suggestions. First, as Serletti et a. (ref. 14) and Nelson et al (ref. 16) used the similar protocol, and we discussed about it from your second comment. Second, since ‘PMID: 2810204’ dealt with only two cases of thrombolysis using urokinase, it is difficult to make meaningful statistical comparisons of the systemic side effects due to urokinase. Finally, we think that the limitation section is not appropriate as a place to declare plans for the next study. When the data of the authors are further accumulated, a more meaningful research presentation will be made. We also look forward to the next research presentation through PLOS one.**

• The conclusions are not very satisfying: your protocol aims at making free flap salvage safer by reducing the overall urokinase dosage and by avoiding systemic circulation unlike what has been proposed in previous protocols. This should be stressed appropriately.

**ANSWER: We appreciate your comments. We discussed about it in the second comment. Then, we corrected conclusion more precisely as above-mentioned in the red font.**

**The free flap can be effectively and safely salvaged, even in delayed salvage cases, using intra-arterial urokinase infusion without systemic hemorrhagic complications and results in successful salvage and total flap survival.**

- **The free flap can be effectively and safely salvaged, even in delayed salvage cases, using high-dose intra-arterial urokinase infusion within a short period of time without systemic circulation resulting in the avoidance of systemic hemorrhagic complications and results in successful salvage and total flap survival.**

• Just as a minor observation: while the contents are very understandable, there are a few English language mistakes spread in the manuscript (i.e. “Intra-arterial urokinase infusion is an effective micro-thrombolysis in delayed salvage cases” in the first sentence of your Discussion: do you mean “an effective thrombolytic agent” or “thrombolytic strategy”?). I would recommend that the manuscript is spell-checked by a native speaker.

**ANSWER: Thank you for the suggestion. We changed it to ‘micro-thrombolytic strategy’. And according to your advice, we spell-checked the manuscript again.**
