# Supplementary material for: Safe and effective thrombolysis in free flap salvage: Intra-arterial urokinase infusion
Source: PLoS One. 2023 Mar 13;18(3):e0282908. doi: 10.1371/journal.pone.0282908 (PMC10010546; doi:10.1371/journal.pone.0282908)
Supplement: Attachment — Submitted filename: Response to Reviewers.docx [file pone.0282908.s004.docx]

Reviewer #2: This is the first revision to a previously assessed manuscript. Your manuscript has been marginally improved, however I must insist on some of the points raised in the previous round. First and foremost, there are no restrictions on word count. Thus, I encourage you to present and discuss some of your findings in a more complete manner that can involve the reader for a more in-depth analysis of your work.

**We would like to thank you for the valuable comments and feedback on our manuscript. We have taken your suggestions into consideration and made the necessary revisions to improve the quality and clarity of the paper. We believe that the paper is now ready for publication and would like to request that it be reconsidered for publication in *PLOS one*. Thank you for your time and consideration.**

• Tables 1 through 4 have been implemented. The data needs to be presented more pertinently in the discussion. You need to address that with a significant p-value threshold set at 0.05, none of the elements described in Table 2 (i.e. site of thrombosis, time to re-exploration and dose of urokinase infusion) were associated with successful flap salvage, which is very significant, potentially limiting the utility of your findings.

**ANSWER: Thank you for your feedback on the tables we presented. We understand that Table 2 only includes only surgical details, while Table 3 includes comparable descriptions of surgical details including variables of Table 2 with a significant p-value threshold set at 0.05. Then, to avoid duplicate entries, it is better to obtain the necessary information from table 3.**

• I believe that Table 4 adds very little value to the manuscript, and should be omitted. Just specify in the Results section of your manuscript that only 2 cases (12.5%) of donor-site hematoma were reported in your series of 16 patients. Additionally, please specify in which types of flaps those occurred.

**ANSWER: Thank you for the comment. We have omitted Table 4 and reported its contents in the results section.**

• The rationale for your study is still not very clear: while your implementations in red font in the introduction are helpful, I believe that there is more that needs to be said in the discussion. Add a section in which you specify how your protocol implements a safe thrombolysis method that overcomes the high risk of systemic side effects from using urokinase, which previously made it an obstacle to its use. This is because free flap surgery has many bleeding sites on the donor and recipient sites. Therefore, as in this study, it is a great advantage that high-dose urokinase can be used in a short period of time without systemic circulation. The novelty of this protocol is that it allows much higher doses of urokinase compared to what can be given with systemic administration, as it can reach up to 600,000 IU/hour because infusion time is about 10 minutes. The use of more than 500,000 IU of urokinase per day has been proven to have systemic side effects such as intracranial hemorrhage, hematochezia, and gross hematuria.

**ANSWER: We appreciate your advice that will enrich our manuscript. We added that description with the red font in the discussion section. This will definitely contribute to the overall quality of our work.**

**:** Intra-arterial administration of urokinase with external drainage has been demonstrated to be an effective method for delivering excessive doses of the thrombolytic agent within a short time frame. This approach offers the advantage of bypassing systemic circulation, reducing the risk of systemic hemorrhagic complications. In free flap surgery, where extensive bleeding can occur at both the donor and recipient sites, the use of thrombolytic agents is crucial to minimize the risk of bleeding-related complications and improve surgical outcomes. In this study, the infusion rate of urokinase has been reported to reach up to 600,000 IU per hour, with a typical infusion time of 15 minutes. While high doses of urokinase can be effective in treating thromboembolic disorders, it is important to note that doses exceeding 500,000 IU per day may increase the risk of systemic adverse effects, including intracranial hemorrhage, hematochezia, and gross hematuria.

• I acknowledge the implementations in blue font, which are appropriate. However, regarding your figures, you admitted that out of 581 flaps performed between January 2013 and July 2019, you have experienced “much more thrombotic events than 16 cases included in this study”. While this shows scientific integrity and should be commended, it should also be stated explicitly in the manuscript, as the message conveyed from the manuscript in its current form suggests that only 16 cases of flap-related vascular compromised have been reported. It would greatly benefit the manuscript if you implemented this aspect in the Methods section of your manuscript. If possible, you should state what percentage of flaps had vascular compromise needing re-exploration, what flap survival rate you had in the past and if these figures were related to a learning curve of the surgeon(s) performing the flaps.

**ANSWER: We understand your concerns and take your comments seriously. Unfortunately, however, the incompleteness of the data in the early days of free flap surgery has made it difficult to fully satisfy your expectations to reveal the rates of re-exploration and flap survival. Instead, we have made changes to the corresponding part of the method to make the indication of the use of urokinase more clear. We used urokinase in the delayed revision cases when the time to revision was over 24 hours, and we have expressed it more clearly in the revised section of abstract, method, discussion, and conclusion (with blue font).**

**Inserted in abstract**

**:** Thrombolysis with urokinase infusion was administered as salvage treatment for patients who experienced flap compromise more than 24 hours after free flap surgery.

**In method**

: Pharmacological thrombolysis using urokinase was performed in the case of venous thrombus or venous insufficiency even if it was not thrombosis, regardless of the presence or absence of arterial thrombus in the revision performed 24 hours after flap surgery

- In cases where flap compromise occurred more than 24 hours after free flap surgery, thrombolysis with urokinase infusion was administered for salvage treatment regardless of the presence or absence of arterial thrombus, in the presence of venous thrombus or venous insufficiency.

**In discussion**

: Furthermore, urokinase injection is an effective method with a relatively high success rate of revision more than 24 hours after free flap surgery.

**In conclusion**

: The free flap can be effectively and safely salvaged, even in delayed salvage cases more than 24 hours after primary surgery, using high-dose intra-arterial urokinase infusion within a short period of time without systemic circulation resulting in the avoidance of systemic hemorrhagic complications and results in successful salvage and total flap survival.

• It would also be useful if you could discuss whether the salvage protocol you established has affected flap survival rate on the long term, and if so in what way.

**ANSWER: Thank you for the comment. In a retrospective analysis of our institution's medical records, there was no significant difference observed between the short-term and long-term survival rates of free flap. The results of this study suggest that the survival rate reported can be considered equivalent to the long-term survival rate. It is commonly observed in the literature that the survival rate of free flap is reported using similar tone of an argument, leading to an expectation of uniform understanding among readers.**

• Tables 1, 2 and 3 convey the data accurately. However, in the Methods section of your manuscript, the statement “The thrombotic sites were artery in 6 cases and vein in 15 cases.” should be amended by additionally featuring how one case experienced arterial thrombosis with decreased venous drainage, but not venous thrombosis.

**ANSWER: According to your advice, we corrected it under red shade.**

The thrombotic sites were artery in 6 cases and vein in 15 cases.

- **6 cases had arterial thrombosis and 15 cases had venous thrombosis. To clarify, 5 cases presented with both arterial and venous thrombosis, while 10 cases had only venous thrombosis and 1 case had only arterial thrombosis.**

• Another statement in the Methods section of the manuscript “13 (81.3%) flaps survived completely, but 2 cases among them experienced partial necrosis and secondary healing.” still needs to be rephrased in the following manner: from the initial 16 cases, 11 survived completely while 2 had partial necrosis and 3 were totally lost despite the salvage attempt.

**ANSWER: We appreciate your correction. We corrected that phrase under yellow shade.**

13 of 16 (81.3%) flaps survived completely, but 2 cases among them experienced partial necrosis and secondary healing.

- **in a study of 16 patients undergoing flap surgery, 11 flaps were found to have survived completely, while 2 flaps experienced transient partial necrosis, and 3 were lost despite salvage efforts. In other word, 81.3% (13 of 16) of flaps survived.**

• Regarding post-operative administration of heparin, the implementation in brown font was adequate but you failed to mention whether the dosage was the same for all patients of whether it was calculated according to patient’s weight. How many hours is anticoagulation regimen started after salvage surgery? Finally, do you believe that post-operative anticoagulation might concurrently be responsible for bleeding events i.e. donor site hematomas? If so, discuss this and compare your regimen to Serletti et al. (ref. 14) where no post-operative anticoagulation was used.

**ANSEWR: Thank you for the comment. We wanted to draw your attention to a point that was mentioned in the green-shaded section of our previous manuscript. We think you will understand if you look again. The use of heparin is measured 3 times a day while performing continuos infusion, setting the target aPTT range of 30 to 50 seconds. We found that this method was more accurate in monitoring blood coagulation status than heparin dosage based on body weight. In this revised manuscript, we will leave it in the same green shade so that it can be easily found.**

For postoperative management, heparin was administered to prevent recurrent thrombus formation for 7 days and adjusted while monitoring activated partial thromboplastin time (aPTT) range of 30 to 50 seconds 3 times a day.

**We also wanted to mention that we came across a study by Serlatti's group, which demonstrated an excellent method for thrombolysis that does not use heparin. Although this method is noteworthy, since we have adopted the method of using heparin after thrombolysis, we will leave it undeleted for integrity. Nonetheless, we did not observe any systemic hemorrhagic complications after surgery, which suggests that heparin use is also safe when monitored with aPTT in the range of 30 to 50 seconds.**

• Since your time to re-exploration was considerably shorter than other studies, I believe that a concise statement on the monitoring technique you used could serve your manuscript well.

**ANSWER: Thank you fot the advice. We added it under purple shade in the section of Method.**

The flap was meticulously monitored for any clinical signs of vascular compromise, including color, temperature, capillary refill rate, skin tension, turgor, and Doppler sound, every hour for the first 24 hours, every 2 hours for up to 48 hours, and every 4 hours for up to 120 hours postoperatively. In addition, portable ultrasonography was used to evaluate the blood flow of pedicles, which provided an objective assessment of the pedicle's perfusion and enabled early detection of potential thrombotic changes that may not be evident on clinical examination or Doppler ultrasound alone.

• While you emphasized that gentle manipulation of vessel during microsurgery is important, you did not clarify which micro-anastomosis technique was used. This is relevant because endothelial trauma is a major cause of vascular compromise for free flaps (PMID: 24706545). This should be addressed concisely in your Discussion.

**ANSWER: According to your comment, we added it with brown font in the section of Discussion**

Gentle manipulation of vessels during micro-anastomosis is crucial to prevent damage to the endothelium and minimize the risk of thrombosis. In our institute, we use a meticulous technique that involves the use of stay sutures placed at 180 degrees along the vessel circumference, with anterior suturing performed first. To assist with optimal exposure, a cottonoid or similar gauze material with adequate frictional force is placed under the blood vessel and used to carefully rotate the vessel by 180 degrees. Hydrostatic dilation is achieved by squirting heparinized saline solution into the vessel lumen under pressure, which provides adequate exposure of the posterior wall while continuously monitoring the correct positioning of the stitches. This method avoids the need for an Ikuta approximator, which further minimizes the risk of injury to the delicate vascular tissue

• Regarding hemodilution in free flap surgery, hemodynamics play a very pertinent role in vascular compromise. While the authors are entitled to their belief and may not share the practice, I would still recommend discussing it, all the while still sharing why they do not believe in its use. i.e. While hemodilution has been shown to improve tissue oxygenation in ischemic flaps with high flap survival rates, (PMID: 12170063) and has been taken into consideration by some authors when planning free flap surgery, (PMID: 26313824; PMID: 26818324) we do not agree with its use. While it is difficult to apply in patients according to the clinical setting, in our surgery, the ischemic time of free flap is usually less than 60 minutes. According to PMID: 9160132, ischemic time did not affect flap survival or partial loss within 3 hours.

**ANSWER: According to your advice, we added a paragraph about hemodilution and free flap surgery in the section of discussion with purple font. However, PMID: 26818324 was not quoted because it is about a perforator flap with a twisted pedicle and is not about free flap surgery.**

Hemodynamics play a crucial role in vascular compromise during free flap surgery, and while hemodilution has been shown to improve tissue oxygenation due to accelerated erythrocyte velocity with reduced blood viscosity in ischemic flaps with better microcirculation and high flap survival rates in animal model. In addition, normovolemic hemodilution has been found to be a considerably effective method for reducing the incidence of thrombosis and microvascular free flap failure.

• Most of your compromises occurred in ALT flaps. Is this because it represents a workhorse flap in your institution which you perform more commonly than other flaps? If not would you attribute this to a specific risk inherent to the flap or the way you identify perforators? Either way, discuss this finding.

**ANSWER: In fact, since our institution uses ALT flaps most reliably and with a frequency of more than 90%, the compromised rate also appears to be the highest due to the selection bias. We will add this to the limitations of Discusion under grey shade.**

- A selection bias may have been present in our study, as the anterolateral thigh (ALT) flap was the most commonly utilized flap in our institution and thus represented the majority of flaps included in the analysis.
